# Effects of Low-Temperature Hot Isostatic Pressing on Tensile Properties of 316L, AlSi10Mg and GRCop42 Alloys Produced by PBF-LB

**DOI:** 10.3390/ma19122468

**Published:** 2026-06-09

**Authors:** Daniele Cortis, Cristina Giancarli, Claudio Testani, Giuseppe Barbieri, Donato Orlandi

**Affiliations:** 1 Gran Sasso National Laboratory (LNGS), National Institute for Nuclear Physics (INFN), Via G. Acitelli 22, 67100 L’Aquila, Italy; daniele.cortis@lngs.infn.it (D.C.); cristina.giancarli@lngs.infn.it (C.G.); donato.orlandi@lngs.infn.it (D.O.); 2 CALEF Consortium, Italian National Agency for New Technologies, Energy and Sustainable Economic Development (ENEA), Via Anguillarese 301, Santa Maria di Galeria, 00123 Rome, Italy; 3 Italian National Agency for New Technologies, Energy and Sustainable Economic Development (ENEA), Via Anguillarese 301, Santa Maria di Galeria, 00123 Rome, Italy; giuseppe.barbieri@enea.it

**Keywords:** aditive manufacturing, power bed fusion–laser based, hot isostatic pressing, tensile properties, microstructure, 316L, AlSi10Mg, GRCop42

## Abstract

Powder Bed Fusion–Laser Based (PBF-LB) represents the most-used metal Additive Manufacturing technology thanks to its capability of producing high-complexity geometries. The need for industries to define a qualification framework of additive components drew attention to post-processing approaches that can be applied to mitigate or reduce inherent defects. Among these post-processing approaches, Hot Isostatic Pressing (HIP) is recognized as one of the most effective techniques to address these challenges. Among materials employed with PBF-LB, especially in the aerospace sector, 316L stainless steel and the AlSi10Mg aluminum alloy are the most investigated, while among innovative copper alloys, there is GRCop42. Thus, the aim of this paper is to investigate the effects of low-temperature HIP on the tensile properties and microstructure of these materials. For this reason, tensile tests, metallographic analysis and X-ray computer tomography were conducted. The results highlight the influence of low-temperature HIP treatment with respect to the as-built condition. In particular, the Yield and Ultimate Tensile Strength for 316L and GRCop42 clearly improved, while for AlSi10Mg a relevant reduction was detected. However, an unexpected result was the reduction in the GRCop42 elongation that fell from ~10% down to ~2.5%, even though the porosity of the material was reduced to close to zero.

## 1. Introduction

Powder Bed Fusion–Laser Based (PBF-LB) [1] represents the most widely used metal Additive Manufacturing (AM) technology thanks to its capability to produce complex geometries with high precision and the possibility to manufacture a wide range of materials, like steel, aluminum, nickel, copper and titanium alloys. At the heart of the technology is the laser source, which selectively melts a thin layer of metal powder to build parts layer by layer. Due to the nature of this specific process, the components produced may be subject to metallurgical defects such as porosity and cracking, which reduce the material relative density with respect to a cast one. Moreover, the layer-by-layer manufacturing process along a specific direction introduces anisotropies and residual stresses that must be considered, especially during the post-processing of parts and the experimental material characterization. In fact, the presence of metallurgical defects can adversely reduce the tensile strength, acting, for example, as stress concentration sites and leading to premature failure. Commonly, PBF-LB defects can be classified into surface and internal. The first ones, like oxide contamination, balling, spatters, etc., are more detectable and can be removed by means of secondary operation, like machining. On the contrary, the second ones are more difficult to be uncovered and can typically be categorized into porosity due to lack of fusion, gas pores, keyhole and cracks, mainly due to inappropriate laser parameters and process instability [2].

In this scenario, the need for industries (e.g., aerospace, automotive, biomedical, etc.) to create and define a qualification framework of AM components, such as that of ASTM [3], which takes into account processes, materials, design strategies, facilities, and personal qualification, drew attention to post-processing techniques that can be applied to mitigate or reduce the inherent defects of PBF-LB technology. Among the post-processing approaches, Hot Isostatic Pressing (HIP) has been established as one of the most effective techniques to address these quality challenges [4]. HIP consists of placing a metal component inside a closed chamber filled with inert gas (i.e., argon, nitrogen, etc.) at a high temperature and pressure for a defined exposure time. The processing temperature (T) is typically in the range of 0.70–0.90 of the melting temperature (T_m_), while the pressure (p) can change from some ten to hundred MPa depending on the equipment and material [5]. The main outcomes of the HIP process are the increase in the densification, the reduction in metallurgical defects, and the homogenization of the microstructure (i.e., reduction of anisotropy). Also, HIP can act as stress relief and a tailoring technique for material properties, achieving the necessary final mechanical and physical characteristics, like tensile strength, thermal and electrical conductivity (i.e., reducing electron scattering events), corrosion resistance, etc.

Despite its clear advantages, HIP has some limitations. For example, the high processing temperature and pressure can reduce the material tensile strength due to grain coarsening and promote geometry shrinkage and distortion, especially for complex internal structures, making the treated parts different from the original shape. Also, the closure of surface-related defects, which often require further post-processing operations such as machining or polishing, are not removed with HIP treatment and may act as a potential nucleation site for crack initiation. Moreover, the outcomes are strictly related to the material composition (i.e., alloy elements), the PBF-LB initial microstructure and cycle parameters. Finally, HIP is a time-consuming and cost-intensive technology, and the decision between outsourcing and an in-house system must be carefully assessed based on production volumes and the knowledge required [6].

Referring to microstructural changes, HIP plays an important role in grain size modification. Usually, high temperature promotes grain coarsening and the resulting reduction in grain boundaries, which diminishes the capability of materials to resist dislocation movements (i.e., deformation). Among the macroscopic effects, a reduction in hardness and tensile strength and an increase in ductility and fracture toughness can be observed for residual stresses relief [7] and internal porosity healing. The grain boundary migration can be limited, for example, by the presence of precipitates finely dispersed inside the material matrix or by impurities. However, the diffusion of these elements can also be accelerated by high temperatures, thereby reducing the dislocation pinning. Therefore, it is evident that the composition of the material, its state condition, the process parameters and the desired physical properties are all factors that must be taken into account when determining the type of HIP treatment to be applied. Recent studies [8,9] highlight the possibility to preserve a fine microstructure with a low-temperature HIP, i.e., by means of a T < ~0.70 T_m_. This solution allows for detection mitigation while maintaining the high mechanical properties produced by the PBF-LB microstructure.

Among common materials employed with the PBF-LB technology, 316L stainless steel and AlSi10Mg aluminum alloy are the most investigated [10]. First, 316L is a low-carbon austenitic steel (C < 0.03%) that offers excellent weldability, making it one of the most popular and widely used materials in the metal AM thanks also to its very low cost. The low-carbon content and the presence of molybdenum (~2–3%) make it resistant to intergranular corrosion. The high thermal gradient also produces a full-austenitic microstructure that has already been solution-treated, without the need for other heat treatments, except for stress relief before to remove the components from the build platform. Also, the high cooling ensures a very fine grain size, which contributes to its improved mechanical properties compared to the conventional casted material. Concerning AlSi10Mg, the high content of silicon classifies the material inside the aluminum 4xxx series [11]. The presence of silicon increases the flowability and reduces the shrinking during solidification, eliminating hot-cracking brittleness. These properties are essential for casting but also for PBF-LB technology and have contributed to its wide industrial adoption. Finally, among the most innovative copper alloys that are highly processable using PBF-LB technology, there is GRCop42 (Glenn Research Center Copper, Cu-4 wt.% Cr-2 wt.% Nb). It belongs to the Cu-Cr-Nb alloy family, and its great characteristics are largely governed by the presence of a Cr_2_Nb nanoscale secondary phase. The coarsening resistance and stability at high temperatures of Cr_2_Nb (i.e., up to ~800 °C) gives the material high tensile strength, while the copper matrix ensures high thermal conductivity [12,13]. Finally, these alloys can be post-processed after PBF-LB to increase densification and mechanical and physical performance using thermal processes such as HIP.

Thus, the objectives of the research are to investigate the effects of low-temperature HIP cycles on: (i) tensile properties, (ii) microstructure and (iii) defect reduction of the above alloys (i.e., 316L, AlSi10Mg and GRCop42) manufactured with PBF-LB technology. Tensile tests were conducted with a standard universal testing machine, while a microstructures effect evaluation was performed by metallographic analyses (Digital Microscope, VHX-7000N, KEYENCE Corporation, Osaka, Japan). Finally, the reduction in defects was assessed by X-ray computer tomography (CT). The results highlight the clear influence of HIP treatment with respect to the as-built (AB) material condition, showing, for example, an opposing behavior between the AlSi10Mg and GRCop42 alloys due to microstructural changes and the precipitate phases.

## 2. Materials and Methods

### 2.1. Materials Powder

Powders of the three alloys (i.e., 316L, AlSi10Mg and GRCop42) were supplied by Metals4Printing company (Fesitritz im Rosental, Austria). The powders’ Particle Size Distribution (PSD), expressed as D15 and D90 cumulative percentile, was in the range of 15–45 µm for 316L and AlSi10Mg and in the range of 20–63 µm for GRCop42. The powder chemical composition ranges and the measured values (i.e., by ICP-OES standard), as declared by the supplier [14], are reported in Table 1.

### 2.2. Tensile Test Piece Production

**Figure 1:**
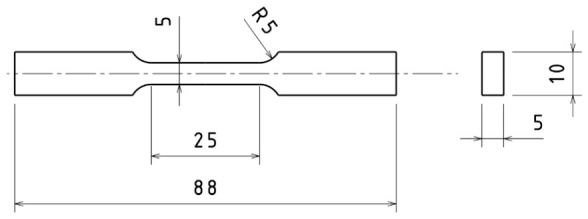
Tensile test pieces’ geometry and dimensions according to ASTM E8/E8M.

Tensile test piece geometry and dimensions (Figure 1) were defined according to the ASTM E8/E8M standard [15]. The production was done at the Additive Manufacturing facility of INFN-LNGS laboratory by means of a standard PBF-LB machine (i.e., MySint100 PM/RM, SISMA, Vicenza, Italy) with open process parameter configuration specifically developed for research and development applications. The machine is equipped with an infrared laser source up to 200 W and a laser spot size of 30 µm, which guarantee high geometrical accuracy. The building platform has a cylindrical shape, with a diameter and height of 100 mm. For this reason, the overall length of the test piece was set to 88 mm and the gauge length to 25 mm. The entire manufacturing process took place in an inert argon atmosphere with a residual oxygen level < 100 ppm (0.1%).

The PBF-LB process parameters, such as Laser Power (*P*), Laser Scanning Speed (*S*), Hatch Distance (*H*) and Layer Thickness (*L*), were selected based on the authors’ previous works [16,17,18] and are listed in Table 2, together with the Volumetric Energy Density (VED), Equation (1), applied by the laser source on the powder bed during the layer-by-layer manufacturing process. These combinations of process parameters guarantee for all three alloys a relative density (ρ) in the range of 98.1% and 99.6%. Only the GRCop42 alloy did not exceed 99% due to the well-known low coefficient of absorption of copper at infrared laser wavelengths (i.e., ~1070 nm) [19]. However, this density level is acceptable and useful for the purposes of the present study.

(1) $$VED=\frac{P}{S\;H\;L}$$

**Figure 2:**
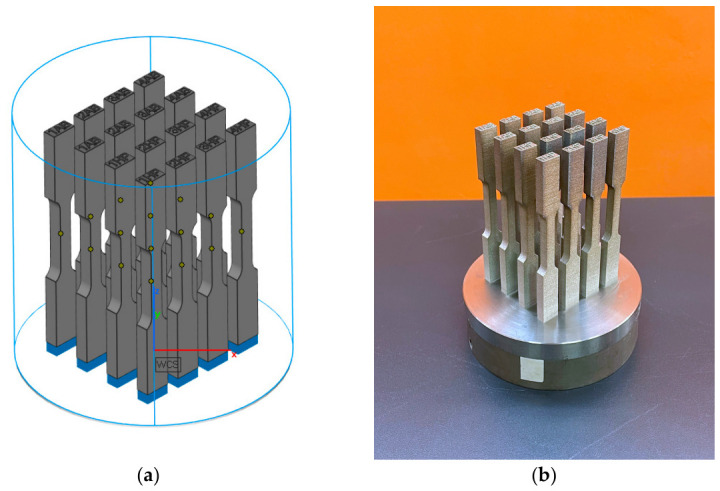
Tensile test pieces production details: (**a**) placement inside the volume of the cylindrical building platform; (**b**) 316L tensile test pieces after the PBF-LB production.

Tensile test pieces were manufactured vertically with the main axis parallel to the building direction of the PBF-LB machine (MySint100 PM/RM, SISMA, Vicenza, Italy), see Figure 2a. The connection to the substrate (i.e., stainless steel for 316L and GRCop42, aluminum for AlSi10Mg) was secured by means of 5 mm of supports, which were removed after the production using electrical discharge machining. Each material production job consisted of 12 test pieces, 6 of which were left in the AB condition, whilst the other 6 underwent HIP treatment. Figure 2b shows an example of the 316L test pieces after the PBF-LB production on the building platform.

### 2.3. Hot Isostatic Pressing Treatment

HIP treatment on three different alloys was done at the laboratories of ENEA’s CALEF consortium. As described in the introduction, HIP treatment is usually performed at a temperature (T) in the range of 0.70–0.90 of the melting temperature (T_m_) to maximize the defect elimination. In this case, the authors chose to carry out a low-temperature HIP treatment to limit the grain coarsening, permitting the reduction in pores and defects (i.e., a full densification) while conserving the fine grain microstructure typical of the PBF-LB manufacturing process. In fact, the low-temperature HIP is a different solution that offers a balance between defect elimination, anisotropy reduction and material tensile strength given by the PBF-LB microstructure.

**Figure 3:**
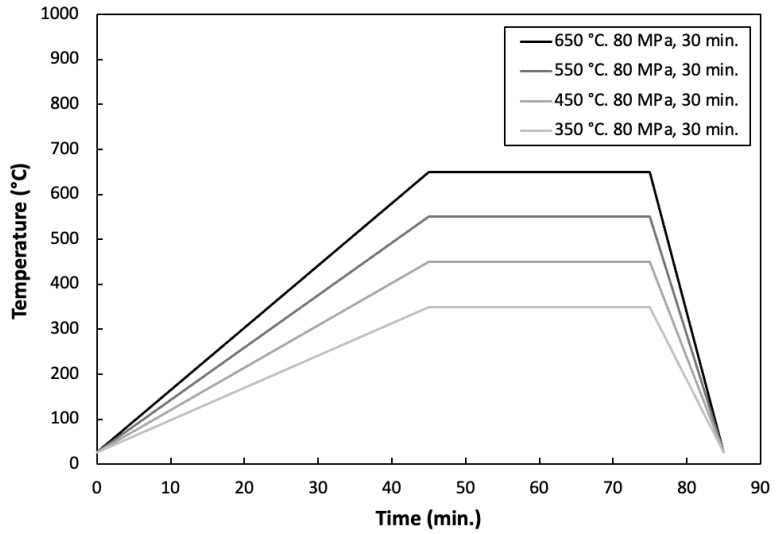
Typical HIP cycles applied for the treatment of 316L, AlSi10Mg and GRCop42 alloys.

Table 3 reports the HIP treatment parameters, such as T, T_m_, T/T_m_, pressure (p) and time (t), that were applied on the three alloys. To assess the effects of the HIP treatment, it was decided to vary only the T across two levels, without exceeding 0.7 of T_m_, while keeping the pressure and time constant. A typical HIP cycle graph is shown in Figure 3, where it is also possible to observe the different temperature gradients applied during the heating and cooling phases.

### 2.4. Material Characterization

Tensile tests were done at room temperature by means of a universal tensile machine (i.e., INSTRON 68FM100, INSTRON, Massachusetts, MA, USA) equipped with a load cell of 100 kN at the Additive Manufacturing facility of INFN-LNGS laboratory. The strains for the evaluation of the Yield Strength (YS), Ultimate Tensile Strength (UTS) and elongation (A) up to rupture were measured by an axial clip-on extensometer (i.e., INSTRON 2630-105, INSTRON, Massachusetts, MA, USA) with a gauge length of 25 mm. Tests were performed in strain-rate control mode with a constant value of 0.00025 s^−1^. Two test pieces were tested for each condition (i.e., AB, HIP#1 and HIP#2), while the others were kept as spare and for the metallographic and CT analysis.

After tensile tests, the material microstructure was evaluated by means of an optical micrograph, realized by means of a high-resolution optical microscope (i.e., VHX7000, KEYENCE, Osaka, Japan). Material samples were polished and etched until the melt pools became visible according to the indication of ASTM E407-07 [20] (i.e., 316L: 9 g NH_4_CuCl_3_ + 150 mL HCl + 45 g FeCl_3_ + 75 mL H_2_O; AlSi10Mg: 2 mL HF + 3 mL HCl + 5 NHO_3_ + 190 mL H_2_C; GRCop42: 2 g K_2_Cr_2_O_7_, 8 mL H_2_SO_4_, 4 drops HCl, and 100 mL water).

At the same time, a CT inspection was performed on some test pieces at the laboratories of the ENEA’s CALEF consortium with a maximum X-ray tube voltage of 450 kV (i.e., XE-L HE, Gilardoni, Lecco, Italy) to evaluate the defect reduction after the HIP#1 and HIP#2 treatments. Data were analyzed by means of VGStudio MAX software version 3.5.2 [21] provided with a CT reconstruction module. The minimum detectable defect size was estimated at 5 µm. The results of the analyses are set out in the next section.

## 3. Results

### 3.1. Tensile Properties

**Figure 4:**
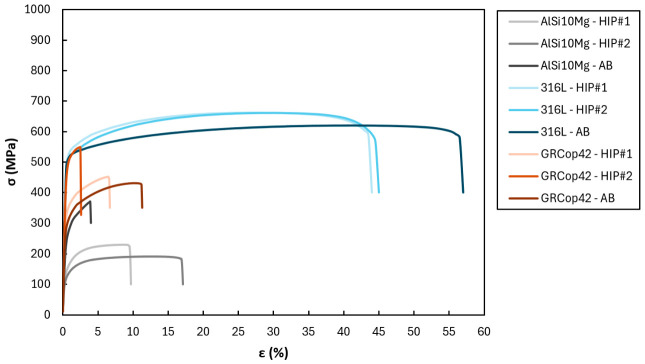
Stress (σ)–strain (ε) curves of 316L, AlSi10Mg and GRCop42 alloys in the AB and HIP conditions.

Tensile properties of the three alloys are reported, in terms of stress (σ) and strain (ε) behavior, in Figure 4, while the YS, UTS and A mean value are listed in Table 4. Concerning the 316L alloy, the HIP#1 and HIP#2 treatments resulted in an increase in the UTS of approximately 7%, from ~610 MPa to ~660 MPa, while the YS remained almost unchanged. On the other hand, the A decreased from above ~50% to ~40–45% (Figure 4). It can be stated that there is no significant difference in the tensile behavior between the HP#1 and HIP#2 treatment.

The case of the AlSi10Mg alloy is completely different. The HIP#1 and HIP#2 treatments progressively reduced the material’s strength from a UTS of ~400 MPa to a value below 200 MPa. The same trend was observed for the YS, while for the A, as expected, a relevant increase was noted from ~4% to a value in the range of ~15–20%. In this case, a change of just +50 °C in the T was enough to significantly alter the material’s properties between the HIP#1 and HIP#2 treatment.

A similar but opposite behavior was observed for the GRCop42 alloy. The HIP#1 and HIP#2 treatments progressively increased the material’s strength from a UTS of ~430 MPa to a value above 500 MPa. As expected, the same trend was observed for the YS, while for the A, a progressive reduction was noted from ~10% to just ~2–3%. Even in this case, there was a significantly change in the material’s properties between the HIP#1 and HIP#2 treatments. Finally, the evaluation of the elastic modulus (E) of the materials confirmed that the value remains constant from the AB and HIP condition: E_(316L)_ = ~190 GPa, E_(AlSi10Mg)_ = ~70 GPa and E_(GRCop42)_ = ~110 GPa.

### 3.2. Material Microstructure

**Figure 5:**
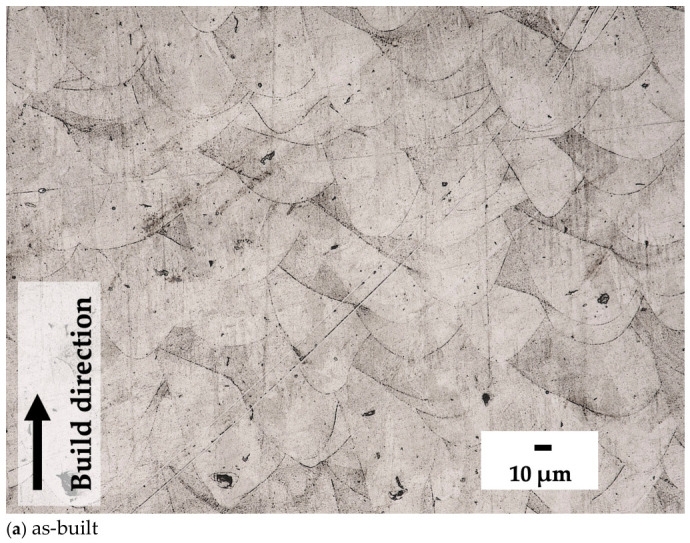

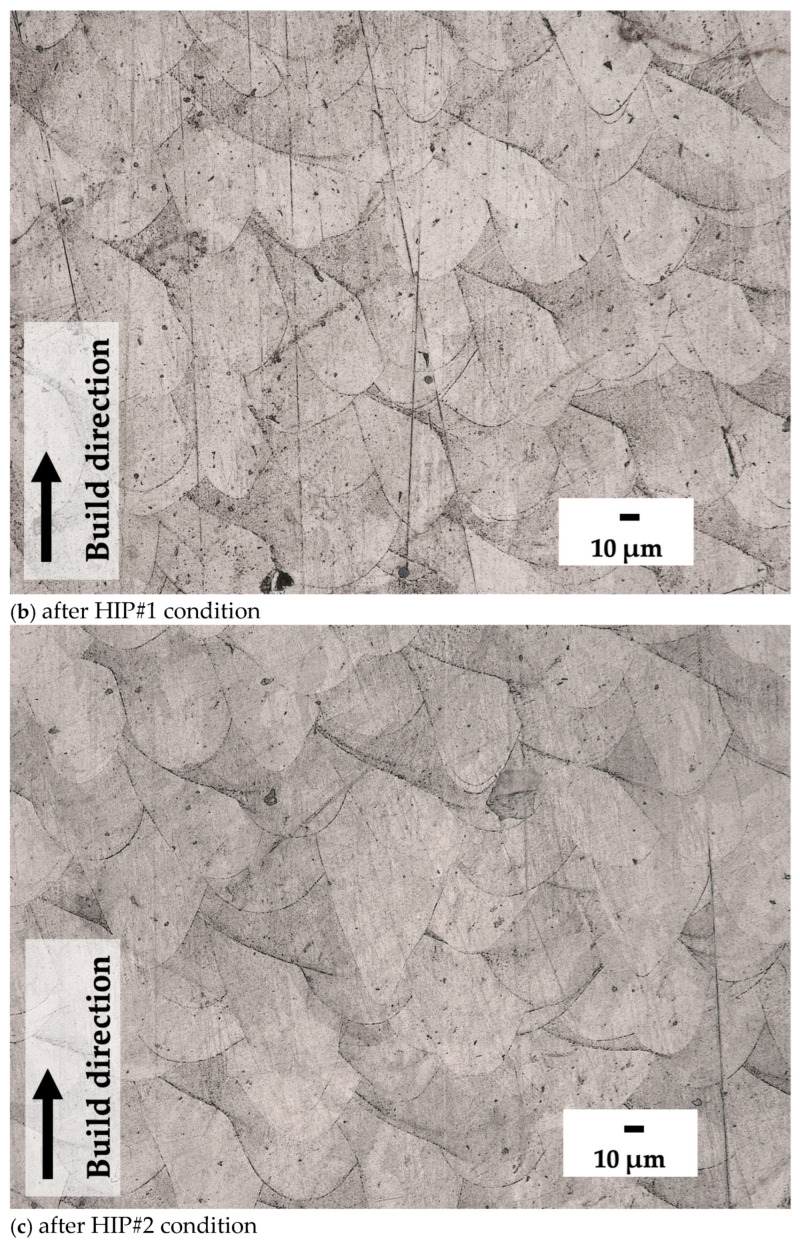
Optical micrograph of the 316L alloy (500×): as-built and after the two HIP cycles. For HIP details, see Table 3.

**Figure 6:**
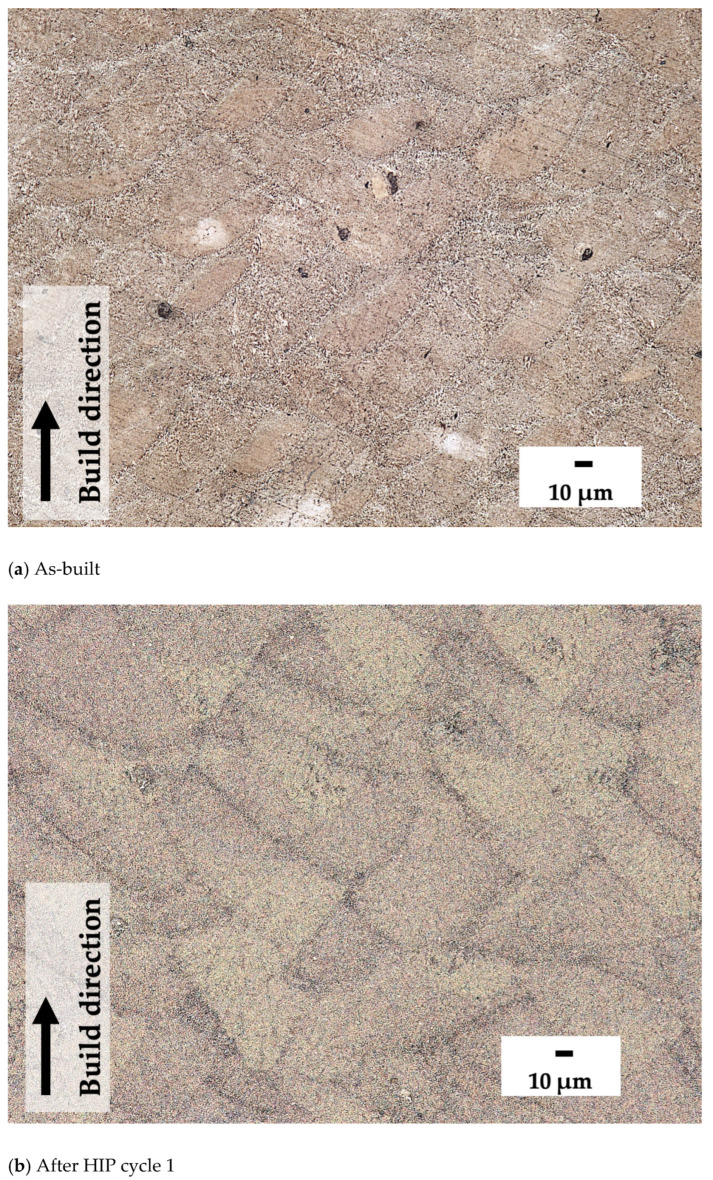

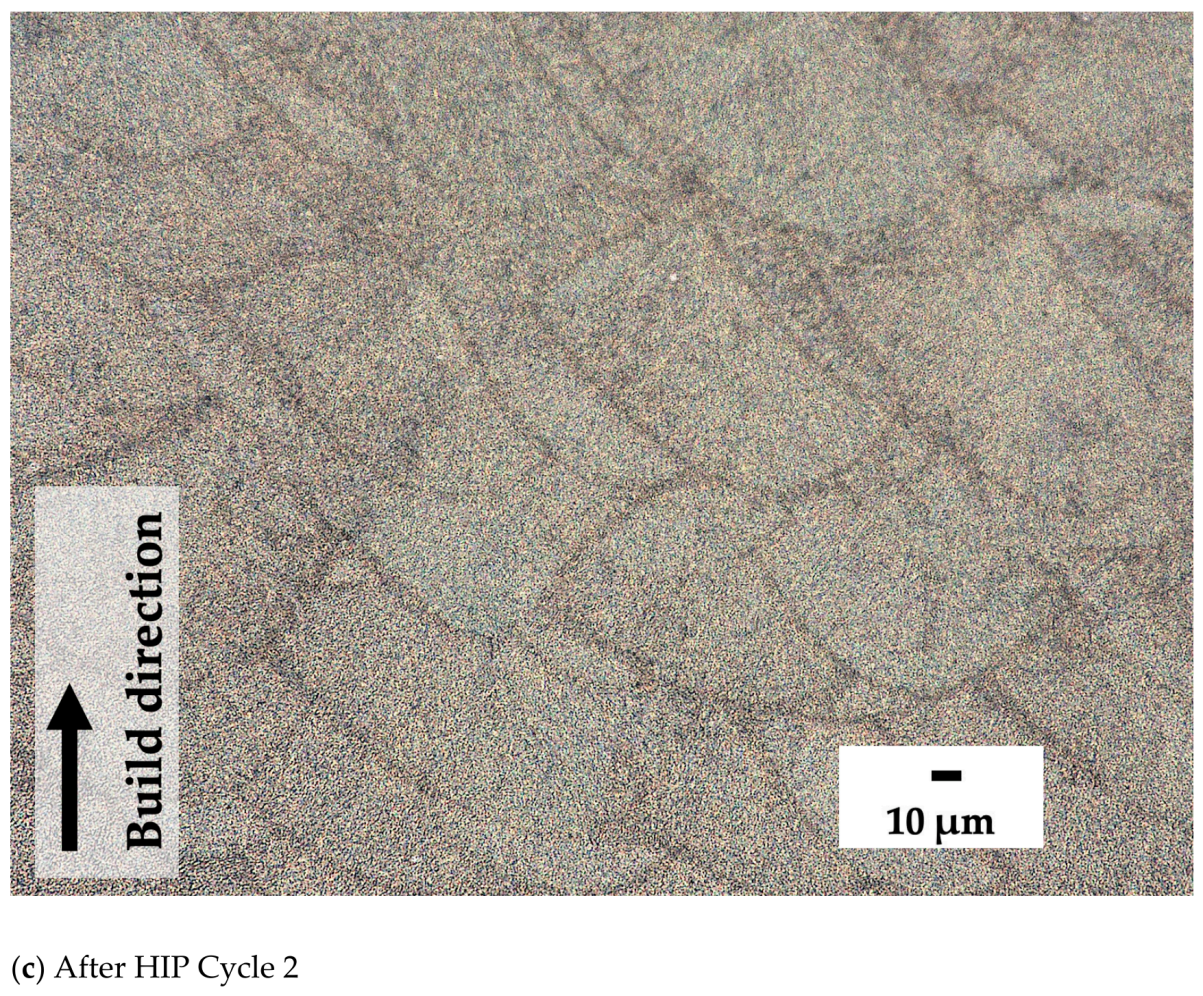
Optical micrograph of the AlSi10Mg alloy (500×): (**a**) AB condition; (**b**) HIP cycle 1 condition; (**c**) HIP cycle 2 condition.

**Figure 7:**
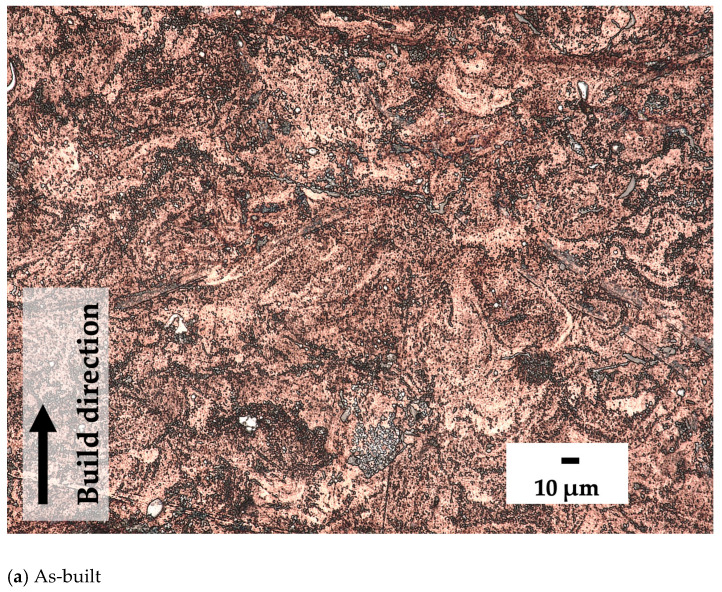

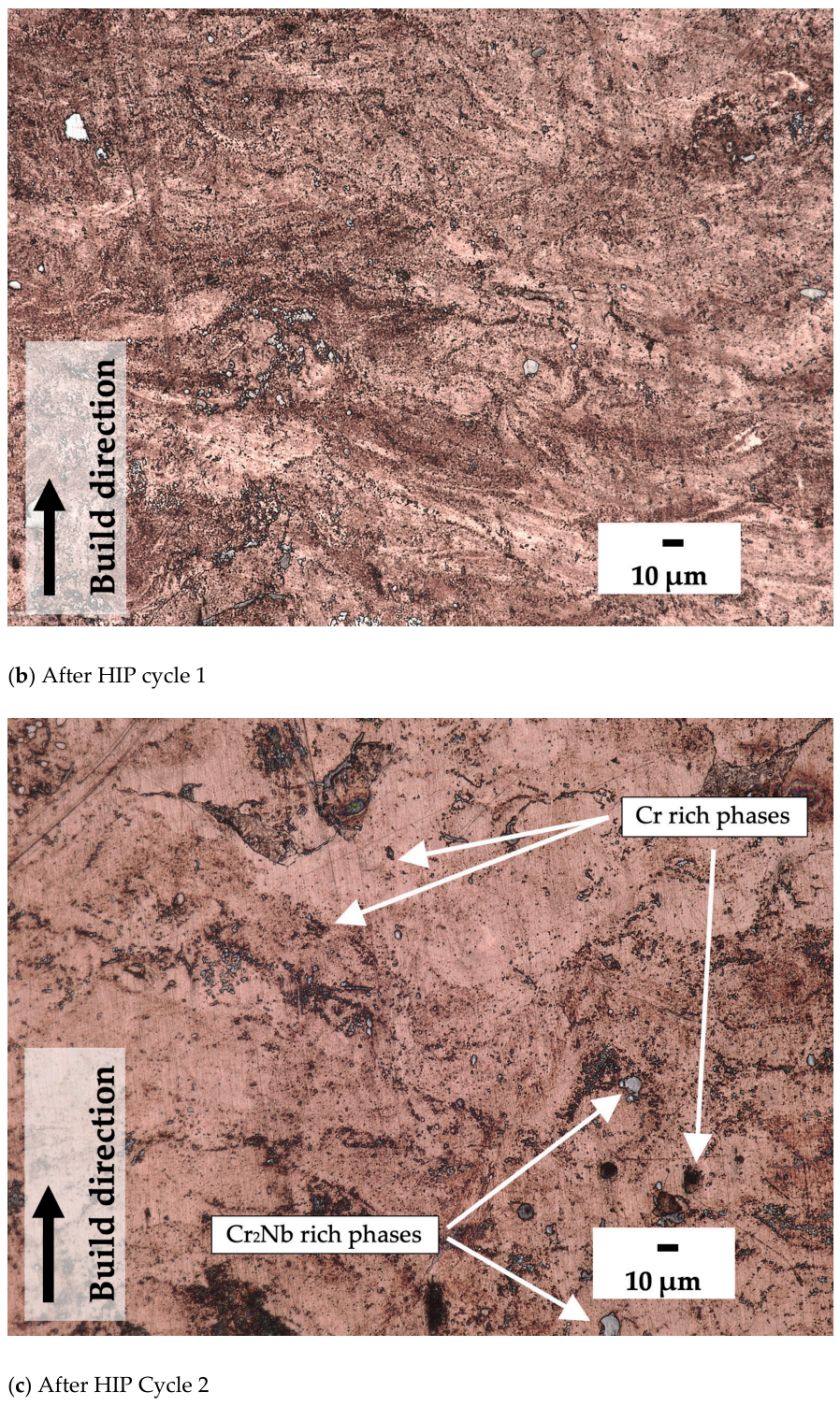
Optical micrograph of the GRCop42 alloy (500×): (**a**) AB condition; (**b**) HIP cycle 1 condition; (**c**) HIP cycle 2 condition. See Table 3. From [22], the grey, darker areas are Cr-rich phases and the lighter ones are phases of Cr_2_Nb [22].

Figure 5, Figure 6 and Figure 7 report the optical micrographs done on the tensile test pieces of the three materials along the PBF-LB building direction, where the melt pool size and overlapping of laser tracks between the different layers can be noticed. Concerning the 316L alloy, no areas with different morphologies were noted (Figure 5); the laser tracks and melt pools appear to be well visible and distributed along the build direction. Similarly, for the AlSi10Mg alloy, no relevant changes in melt pool tracks were observed after the HIP treatments (Figure 6). GRCop42 is a different matter, where the different distribution of Cr_2_Nb precipitates can be highlighted (Figure 7). In particular, the grey areas can be attributed to a Cr- and Nb-rich phase inside the Cu matrix, where the darker ones are Cr and the lighter ones are the phase of Cr_2_Nb [22].

**Figure 8:**
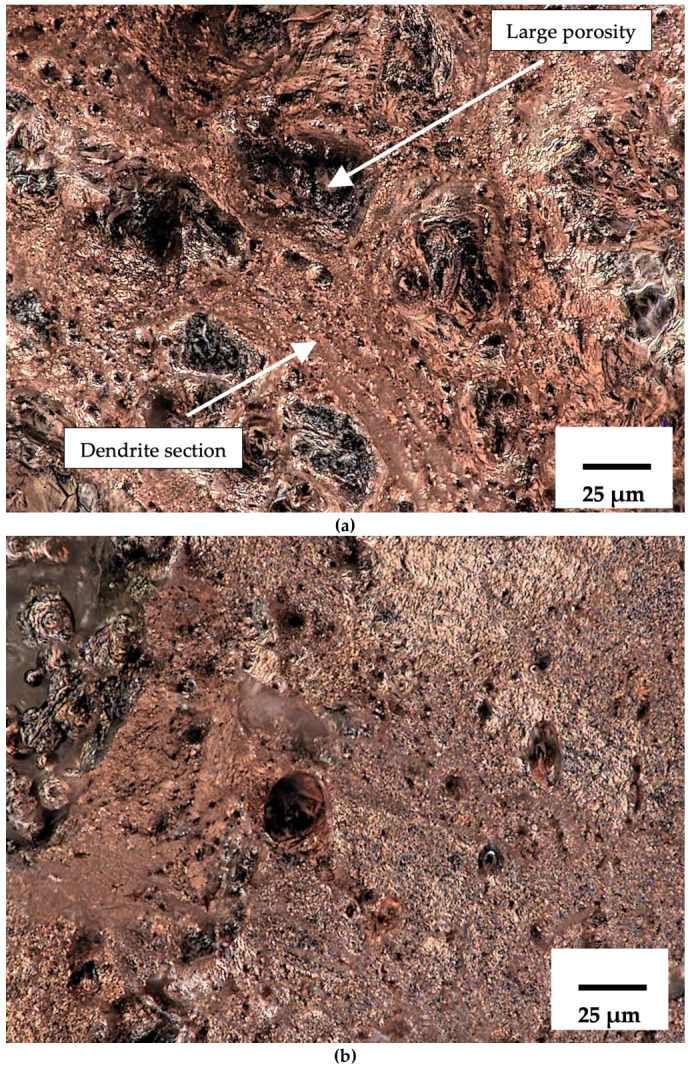
Digital stereo optical micrograph of the GRCop42 alloy: (**a**) before treatment HIP and after (**b**) cycle HIP at T = 550 °C.

Eventually, a CT inspection was done on some tensile test pieces to evaluate the defect reduction after the HIP treatment. Among the treatments, the HIP with the highest temperature was selected (i.e., HIP#2). The results, reported in Table 5, are highlighted, as the HIP can significantly reduce the residual presence of porosity inside the materials, which is difficult to remove by optimizing the PBF-LB process parameters. Also, the CT analysis of the AB condition confirms the relative density estimation done with the Archimedes’ principles for the three alloys (Table 2). In this case, the microporosities were revealed on the surface of the tensile test pieces. An exemplum is shown in Figure 8 for GRCop42 before and after the GRCop-HIP#2 treatment at T = 550 °C.

## 4. Discussion

The ASTM F3184-16 standard [23] suggests specific HIP parameters for the 316L alloy produced by PBF-LB (i.e., T = 1120–1163 °C, *p* ≥ 100 MPa, t = 240 ± 60 min.) to achieve full density, close internal pores, heal micro-cracks, and homogenize the microstructure. However, high temperatures can affect the advantages of the typical microstructure of PBF-LB, reducing the YS and UTS. The literature proposes several studies [24,25] on 316L, most of them using an HIP temperature exceeding the temperature of our investigation (i.e., 550–650 °C). The studies are highlighted because the HIP homogenizes the fluctuation and increases the reproducibility of mechanical properties and fatigue strength with respect to the AB conditions; this is also true for components produced in different locations of the building platform [6], thus reducing the premature failure due to the inherent defects of the material. Another effect to be considered is the precipitation hardening that is activated during the HIP treatments and is evident for the steel and Al alloys [26,27] and especially for the copper alloy, as discussed below. In any case, even if this phenomenon could also be activated by diffusion, we believe that it is always over-imposed to other macroscopic effects such us porosity relief. Generally, the tensile strength in the HIP test pieces is slightly increased with respect to the AB condition, with just a bit of reduction in ductility, confirming the evidence of our study, where 316L experienced modest changes in tensile properties after the HIP#1/2 treatments (Table 4). The microstructure of the AB condition consists of a sub-microstructure of equiaxed sub-grains and columnar structures typical of the PBF-LB production process (i.e., high cooling rate). Each melt pool is composed by this morphology, where grains are aligned along the thermal gradient, resulting in a heterogeneous and anisotropic structure. The HIP process can replace this microstructure, leading to recrystallization and grain growth. At elevated temperatures (i.e., ~1100 °C) atoms have sufficient mobility to migrate along grain boundaries and dislocation networks. The diffusion allows the elimination of melt pool boundaries, porosity, and dislocations, generating a more stable microstructure [5]. Considering low-temperature HIP treatment, the typical PBF-LB microstructure seems to be unaffected by temperatures below 700 °C [28]. This fact is confirmed by the highest hardness and tensile strength of the material treated in this condition, which coincides with its smaller grain size distribution. In fact, a high value of the YS/UTS is always attributed to a finer granular structure and the high dislocation density present in AB materials. Finally, CT inspections confirmed the efficacy of HIP in achieving a complete reduction in defects, thereby contributing to increasing the reproducibility of mechanical properties of the test pieces (Table 5).

Concerning the AlSi10Mg alloy, even with a low-temperature HIP process (e.g., HIP#1), the material shows a significant tensile strength decrease. However, at the same time, the ductility increases as well. The explanation of this behavior was previously reported in the literature for a higher processing temperature, pressure and time (i.e., T = 500 °C, *p* = 100 MPa, t = 120 min.), and it was associated with a drastic change in microstructure from the AB to the HIP condition. The fine dendritic cell microstructure changes to a granular Si precipitate phase [29]. Other studies that investigated a similar temperature range (i.e., T = 200–500 °C, *p* = 100 MPa, t = 120 min.) discovered how the increase in the HIP temperature promotes the Si precipitation, resulting in a break-down of the Si network structure and leading to a coarser precipitate [30]. In the literature it is reported that the heat-affected zone could be the nucleation site of coarse, incoherent Si precipitates where fracture occurs [31]. As a consequence, there is a trade-off region, where the tensile strength decreases with the temperature increase, while thermal and electrical conductivity are improved. In fact, above 400 °C, the Si network structure collapses, coarsening the precipitates. These modifications resulted in a densification of the aluminum matrix and in a reduction of the Si in solid solution, improving thermal and electrical conductivity. In our case, the evidence of the tensile tests results confirms this behavior and the gradual increase in grain size at the detriment of the tensile strength (Table 3), despite the low temperature of HIP#1 (i.e., ~0.59 T_m_). In terms of densification, the CT inspection revealed no apparent defects on the HIP#1/2 test pieces as compared to the analysis carried out on the AB material (Table 4).

Regarding the GRCop42 alloy, the HIP treatment usually results in a reduction in tensile strength but with a relevant increase in elongation with respect to the AB condition [32,33]. Although the temperature and pressure values at which the HIP treatment is carried out are not reported in the literature, as this information is confidential, the process commonly applied to copper alloys involves a temperature in the range of 800–950 °C with a pressure of 100 MPa [34]; however, some studies report higher temperature and pressure (T = ~1075 °C, *p* = ~200 MPa, t = 120–240 min.) [35]. In our case, the low-temperature HIP process, with respect to [33], resulted in the opposite behavior, with a marked reduction in ductility and an increase in tensile strength with respect to the AB condition (Table 4). Two elements must be considered: (i) metallographic examinations did not reveal any grain growth and (ii) literature reports that the Cr_2_Nb secondary phase at ~500 °C starts to lose its stability [32], promoting diffusion without coarsening. This could produce, for example, a greater number of fine nanoprecipitates [36], affecting the ductility of the alloy. In fact, the increase in finer precipitates may have had a strengthening effect on the matrix by means of a dispersion precipitation mechanism with respect to the AB condition but with a correlated reduction of ductility. Eventually, the CT inspection confirmed the efficacy of the HIP#1/2 treatment, even with low pressures (i.e., 80 MPa), in closing porosity. The analysis showed that the residual defects, resulting from the PBF-LB process (i.e., ~2%), were completely eliminated (Table 5).

## 5. Conclusions

In this paper, an experimental investigation of the effects of low-temperature HIP on tensile properties of 316L, AlSi10Mg and GRCop42 alloys are presented. Material properties were investigated with standard tensile tests, while microstructures and defect reductions, after the HIP treatments, were analyzed by means of micrographs and CT inspections. The results revealed that, even with low-temperature HIP treatments (T < 0.7 T_m_), significant changes in material behavior can be observed with respect to the AB condition.

In detail, (i) the tensile properties for AISI 316L always showed an improvement for both HIP#1 and HIP#2. The best results were obtained for HIP#1, with an 8% higher UTS but a reduction in ductility that still always remained higher than 41%. The post-HIPed aluminum alloy AlSi10Mg showed a large extended ductility improvement (elongation improvement higher than 200%) with a consequent and expected yield and ultimate stress reduction. The post-HIPed GRCop42 exhibited more unexpected tensile property modifications: both HIP cycles improved the Yield and Ultimate Stresses, but the alloy exhibited a ductility reduction. This effect was deeply discussed and presented in correlation with aging precipitation effects due to the HIP “thermal treatments”. (ii) According to the microstructure and (iii) defects, a clear reduction in porosity and printing defects was shown for all three studied alloys (316L, AlSi10Mg and GRCop42) manufactured with the PBF-LB technology. The porosity evaluated by optical microscopy was always reduced and in some cases was under the detectable minimum value of 0.0%. The reasons for these microstructure changes for the AlSi10Mg and GRCop42 alloys are related to the behavior and distribution of the secondary phase. For the aluminum alloy, low-temperature HIP promotes the Si precipitation, leading to a coarser microstructure, with the direct consequence of tensile strength decreases. Similarly, for the copper alloy, the HIP treatment could have produced a greater number of fine Cr_2_Nb nanoprecipitates, with the main effects of reduced ductility and a higher tensile strength with respect to the AB condition.

Future research developments will involve: (i) large-scale test campaigns to understand the reproducibility of the HIP treatment on material performance and (ii) the evaluation of the tensile properties at different temperatures to investigate their operational limits.

## Figures and Tables

**Table 1:** Powder chemical composition ranges and measured values of the 316L, AlSi10Mg and GRCop42 alloys.

Chemical Composition					
Material	Element	Min (wt.%)	Max (wt.%)	Measured (wt.%)	PSD
316L	Fe			Balance	15–45 µm
	Cr	16.0	18.0	17.2	
	Ni	10.5	14.0	10.8	
	Mn	0.0	2.0	1.4	
	Mo	2.0	3.0	2.3	
	Si	0.0	1.0	0.9	
	C	0.0	0.03	0.02	
AlSi10Mg	Al			Balance	15–45 µm
	Si	9.0	11.0	9.2	
	Mg	0.2	0.45	0.31	
	Mn	0.0	0.45	0.39	
	Fe	0.0	0.55	0.14	
GRCop42	Cu			Balance	20–63 µm
	Cr	3.1	3.4	3.2	
	Nb	2.7	3.0	2.8	

**Table 2:** PBF-LB process parameters for 316L, AlSi10Mg and GRCop42 alloys.

Process Parameters	316L	AlSi10Mg	GRCop42
Laser Power (*P*)—W	175	175	175
Laser Scanning Speed (*S*)—mm/s	1450	1100	650
Hatch Distance (*H*)—µm	70	75	60
Layer Thickness (*L*)—µm	40	40	60
Volumetric Energy Density (VED)—J/mm^3^	43.1	53.0	74.8
Relative density * (ρ)—%	99.3	99.6	98.1

* Evaluated by Archimedes’ principle on cubic samples of 10 mm side.

**Table 3:** HIP treatment parameters applied for 316L, AlSi10Mg and GRCop42 alloys.

Treatment	T (°C)	T_m_ (°C)	T/T_m_	*p* (MPa)	t (min.)
316L–HIP#1	550	~1400	0.39	80	30
316L–HIP#2	650		0.46		
AlSi10Mg–HIP#1	350	~590	0.59		
AlSi10Mg–HIP#2	400		0.68		
GRCop42–HIP#1	450	~1085	0.42		
GRCop42–HIP#2	550		0.51		

**Table 4:** Tensile test results for 316L, AlSi10Mg and GRCop42 in the AB and HIP conditions.

Material & Condition	YS (MPa)	UTS (MPa)	A (%)	E (GPa)
316L–AB	472.7 ± 21.1	612.4 ± 20.2	53.6 ± 9.1	~190
316L–HIP#1	478.6 ± 4.0	660.8 ± 3.9	41.1 ± 8.1	
316L–HIP#2	474.4 ± 5.1	657.3 ± 11.0	45.6 ± 2.5	
AlSi10Mg–AB	244.0 ± 4.9	376.1 ± 16.4	4.1 ± 0.6	~70
AlSi10Mg–HIP#1	131.2 ± 3.0	226.5 ± 10.2	12.0 ± 6.4	
AlSi10Mg–HIP#2	111.7 ± 5.9	195.4 ± 11.9	17.3 ± 0.2	
GRCop42–AB	277.4 ± 2.3	427.0 ± 10.2	10.2 ± 3.3	~110
GRCop42–HIP#1	311.5 ± 9.9	432.4 ± 53.2	7.1 ± 1.1	
GRCop42–HIP#2	461.2 ± 41.4	533.6 ± 43.0	2.5 ± 0.1	

**Table 5:** CT inspection data and results for 316L, AlSi10Mg and GRCop42 alloys.

Material	X-Ray Voltage (kV)	Pores (%), AB	Pores (%), HIP#2
316L	200	0.76	0.03
AlSi10Mg	200	0.00	0.00
GRCop42	150	2.19	0.00

## Data Availability

The original contributions presented in this study are included in the article. Further inquiries can be directed to the corresponding author.

## Abbreviations

The following abbreviations are used in this manuscript:

PBF-LB	Power Bed Fusion–Laser Based
AM	Additive Manufacturing
GRCop42	Glenn Research Center Copper (Cu-4 wt. Cr-2 wt.% Nb)
AB	As-Built
HIP	Hot Isostatic Pressing
YS	Yield Strength
UTS	Ultimate Tensile Strength
CT	Computer Tomography
